# Effects and Mechanisms of Tea and Its Bioactive Compounds for the Prevention and Treatment of Cardiovascular Diseases: An Updated Review

**DOI:** 10.3390/antiox8060166

**Published:** 2019-06-06

**Authors:** Shi-Yu Cao, Cai-Ning Zhao, Ren-You Gan, Xiao-Yu Xu, Xin-Lin Wei, Harold Corke, Atanas G. Atanasov, Hua-Bin Li

**Affiliations:** 1 Guangdong Provincial Key Laboratory of Food, Nutrition and Health, Department of Nutrition, School of Public Health, Sun Yat-Sen University, Guangzhou 510080, China; caoshy3@mail2.sysu.edu.cn (S.-Y.C.); zhaocn@mail2.sysu.edu.cn (C.-N.Z.); xuxy53@mail2.sysu.edu.cn (X.-Y.X.); 2 Department of Food Science & Technology, School of Agriculture and Biology, Shanghai Jiao Tong University, Shanghai 200240, China; weixinlin@sjtu.edu.cn (X.-L.W.); hcorke@sjtu.edu.cn (H.C.); 3 The Institute of Genetics and Animal Breeding, Polish Academy of Sciences, Jastrzębiec, 05-552 Magdalenka, Poland; atanas.atanasov@univie.ac.at; 4 Department of Pharmacognosy, University of Vienna, 1090 Vienna, Austria; 5 Institute of Neurobiology, Bulgarian Academy of Sciences, 23 Acad. G. Bonchev str., 1113 Sofia, Bulgaria

**Keywords:** tea, bioactive compounds, polyphenols, EGCG, cardiovascular diseases, mechanisms

## Abstract

Cardiovascular diseases (CVDs) are critical global public health issues with high morbidity and mortality. Epidemiological studies have revealed that regular tea drinking is inversely associated with the risk of CVDs. Additionally, substantial in vitro and in vivo experimental studies have shown that tea and its bioactive compounds are effective in protecting against CVDs. The relevant mechanisms include reducing blood lipid, alleviating ischemia/reperfusion injury, inhibiting oxidative stress, enhancing endothelial function, attenuating inflammation, and protecting cardiomyocyte function. Moreover, some clinical trials also proved the protective role of tea against CVDs. In order to provide a better understanding of the relationship between tea and CVDs, this review summarizes the effects of tea and its bioactive compounds against CVDs and discusses potential mechanisms of action based on evidence from epidemiological, experimental, and clinical studies.

## 1. Introduction

Cardiovascular diseases (CVDs), a group of disorders of the heart and blood vessels, mainly include coronary heart disease (CHD), stroke, heart failure, hypertensive heart disease, rheumatic heart disease, etc. As reported by the World Health Organization (WHO), CVDs are the leading causes of death globally and were responsible for 17.9 million deaths in 2016, accounting for 31% of all global deaths [1]. The proven risk factors of CVDs include unhealthy diet, tobacco consumption, physical inactivity, and harmful use of alcohol [2]. Among these risk factors, diet is suggested to be the most adjustable factor in preventing CVDs. Many studies have shown that fruits, vegetables, cereals, spices, nuts, and mushrooms can prevent CVDs [3,4,5,6,7,8,9,10,11]. Moreover, several studies have indicated that tea and its bioactive components can prevent and treat CVDs as well as improve cardio-metabolic health [12,13].

**Figure 1:**
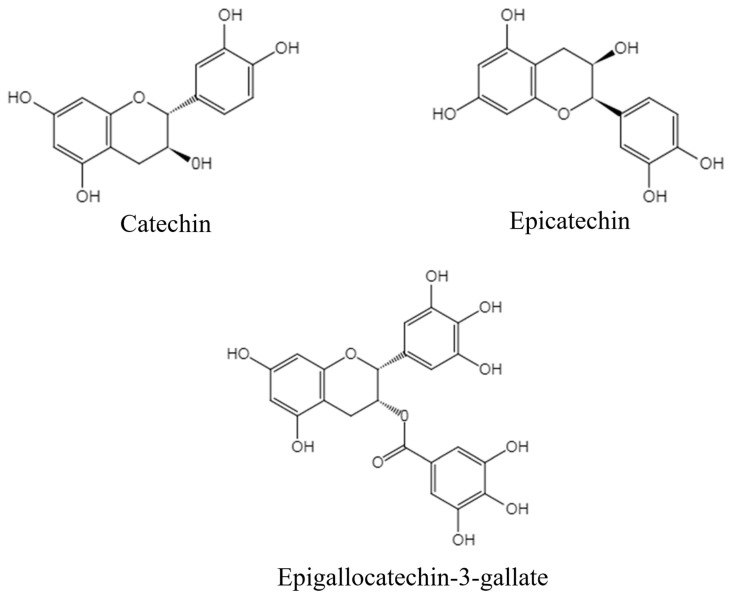
The chemical structures of main catechins in tea associated with cardiovascular disease (CVD) protection.

Tea is the second most consumed beverage worldwide and has a long drinking history of over 2000 years [14]. Tea contains abundant bioactive compounds, which possess favorable effects against many diseases, such as CVDs, obesity, diabetes, liver diseases, and cancers [15,16,17,18,19,20,21]. Numerous epidemiological studies have demonstrated that tea consumption is reversely associated with CVD risk [12,13,22,23]. In addition, in in vitro and in vivo experimental studies, tea and its bioactive components, mainly epicatechin, catechin, and epigallocatechin-3-gallate (EGCG) (Figure 1), have been found to be effective in preventing CVDs, with the mechanisms mainly including lowering blood lipid, ameliorating ischemia/reperfusion injury, attenuating oxidative stress, enhancing endothelial function, relieving inflammation, and protecting cardiomyocyte function [14,24]. Furthermore, clinical trials have also revealed the beneficial effects of tea and its bioactive compounds against CVDs [25,26].

In order to provide a better understanding of the relationship between tea and CVDs, we therefore searched the recent epidemiological, in vitro and in vivo experimental, and clinical studies from the last five years from the Web of Science Core Collection and PubMed databases based on keywords in the title and abstract, including tea, cardiovascular diseases, heart diseases, heart failure, hypertensive heart disease, rheumatic heart disease, and myocardial infarction. The literature types were mainly article and review papers, while meeting abstracts were excluded. This paper provides a comprehensive and updated review on the effects of tea and its bioactive compounds against CVDs, with special attention paid to the relevant mechanisms.

## 2. Epidemiological Studies

Several epidemiological studies have reported that tea consumption has a protective effect against CVDs. A meta-analysis indicated that green tea consumption could significantly reduce the risk of CVDs, and the odds ratio (OR) of myocardial infarction for those drinking 1–3 cups/day of green tea was 0.81 (95% CI: 0.67–0.98) compared with those drinking less than 1 cup/day [12]. Data from the Japan Public Health Center-based prospective study found that consumption of green tea could reduce the risk of heart disease in both women and men, and specifically, that the effect is marginal for decreasing the risk of death from heart disease in non-smoking men [27]. The results from a Chinese cohort study including 165,000 adult men also revealed that habitual green tea drinking was inversely associated with CVD death risk, and the hazard ratios (HR) were 0.93 (95% confidence interval (CI): 0.85–1.01) for ≤5 g/day, 0.91 (95% CI: 0.85–0.98) for 5–10 g/day, and 0.86 (95% CI: 0.79–0.93) for >10 g/day [22]. Additionally, two prospective cohort studies found that drinking green tea could reduce the risk of CVD death with HR 0.86 (95% CI: 0.77–0.97) in middle-aged and elderly Chinese adults [28]. In a Netherlands cohort study, tea consumption was found to be remarkably and nonlinearly associated with the decreased CVD risk in men, with those drinking 2–3 cups/day possessing the lowest HR (0.72, 95% CI: 0.57–0.91) [29]. In addition, a Dongfeng-Tongji cohort study found that green tea consumption could reduce the risk of CHD (HR = 0.89, 95% CI: 0.81–0.98) in the middle-aged and older Chinese population [30]. Furthermore, evidence from the Multi-Ethnic Study of Atherosclerosis conducted on white, Chinese-American, black, and Hispanic populations showed that habitual tea drinking (≥1 cup/day) could inhibit the progression of coronary artery calcification, which led to a decreased cardiovascular event incidence, with HR 0.71 (95% CI: 0.53–0.95), and compared to other race/ethnicity groups, the Chinese-American group had a higher tea consumption and lower incidence of cardiovascular events [31].

Notably, the bioactive compounds in tea also exhibited cardiovascular protective effects in some epidemiological studies. A dose-response meta-analysis regarding flavonoids mainly from tea revealed that flavonoids, such as flavonols, flavones, and flavanones, showed strong effects on reducing CVD risks in a dose-dependent manner, and an increase of 100 mg/day exerted a linear reduced risk of 4% CVD mortality [20]. In addition, the intake of flavonoids from tea and other food was found to be inversely related to CVD mortality and the relevant HRs were 0.34 (95% CI: 0.17–0.69) for data from United States Department of Agriculture (USDA) and 0.32 (95% CI: 0.16–0.61) for data from Phenol-Explorer database [32]. Besides, it has been demonstrated that the Polish population is characterized by a high polyphenol intake, and interestingly, most of the polyphenols are derived from tea and coffee [33]. Subsequently, it has been demonstrated that a higher intake of tea in this population was inversely associated with the risk of cardio-metabolic events [13,34]. In a prospective cohort study with 774 Dutch men aged 65–84 years, epicatechin was found to be associated with a reduced CVD mortality in men with CVDs (HR = 0.54, 95% CI: 0.31–0.96) [23]. Moreover, a high intake of catechins was inversely associated with the risk of CVDs and the HR for a 1-point increment of 10 mg/day was 0.98 (95%CI: 0.96–0.99) in the Nutrinet-Santé French cohort [35]. Furthermore, a prospective, nested case-control study conducted on middle-aged Japanese men found that high serum levels of EGCG could decrease the risk of stroke in non-smoking men, with adjusted OR 0.53 (95% CI: 0.29–0.98) for the highest EGCG level compared with the non-detectable one [36].

Epidemiological studies have indicated that tea consumption could ameliorate cardiovascular risk factors. Hypertension is a major risk factor in CHD and total stroke [37]. The results from the Observation of Cardiovascular Risk Factors in Luxembourg study showed that daily consumption of 100 mL of tea decreased the systolic blood pressure (SBP) by 0.6 mmHg and pulse pressure by 0.5 mmHg [38]. In another study, a cross-sectional study conducted on a rural elderly population in Jiangsu, China, found that tea consumption was significantly and inversely associated with diastolic blood pressure (DBP) (coefficient = −0.74, *p* = 0.003), and frequent tea drinking could reduce the risk of hypertension with OR 0.79 (95% CI: 0.65–0.95), *p* = 0.011 [39]. Moreover, in a longitudinal study conducted on 80,182 Chinese individuals (49 ± 12 years of age), regular tea drinking was found to inhibit the decrease of the serum high-density lipoprotein cholesterol (HDL-C) level in men aged 60 or older, which could reduce the risk of CVDs because a low concentration of HDL-C was suggested to be responsible for high risk of CVDs [40,41]. However, a case-control study using data from INTERHEART China found that habitual tea drinking would increase the risk of acute myocardial infarction, with OR 1.29 (95% CI: 1.03–1.61) for 4 cups/day tea drinkers compared with tea nondrinkers [42]. This inconsistent result may be due to the racial/ethnic factor or the different tea bioactive compound profiles.

Overall, epidemiological studies from Japan, China, the Netherlands, Luxembourg, France, America, and Poland have suggested a favorable role of tea and its bioactive compounds in reducing the risk of CVD incidence and mortality, although a few studies reported that tea could not protect against CVDs. The results of the epidemiological studies are summarized in Table 1.

## 3. Experimental Studies

Increasing in vitro and in vivo experimental studies indicate that tea and its bioactive compounds possess cardiovascular protective effects, including lowering blood lipids, ameliorating ischemia/reperfusion injury, protecting endothelial function, protecting cardiomyocyte function, reducing oxidative stress, and alleviating inflammation, which are discussed below highlighting the relevant mechanisms.

### 3.1. Lowering Blood Lipids

Hyperlipidemia plays a key role in the development of atherosclerosis, and is a vital risk factor for cardiovascular diseases, which can be characterized by changes in the profile of serum lipids, including high triglyceride (TG) level, high cholesterol level, and low HDL-C level [43]. Treatment of male hamsters with mixed extracts of green tea, cocoa, coffee, and garcinia for 6 weeks was found to be effective in lowering serum TG, total cholesterol (TC), low-density lipoprotein-cholesterol (LDL-C), and hepatic TG and cholesterol in a dose-dependent manner [44]. Furthermore, combining the use of green tea extract and eriodictyol could suppress the mRNA level of 3-hydroxy-3-methylglutary-coenzyme A reductase (HMGCR) and 3-hydroxy-3-methylglutary-coenzyme A synthase (HMGCS), and increase the level of LDL receptor, leading to a lowered cholesterol level in male C57BL/6J mice fed with high-fat and high-sucrose diets [45]. Matcha, a kind of powdered green tea, could down-regulate TC, TG, and LDL-C levels, increase HDL level, decrease the serum glucose level, and elevate the superoxide dismutase (SOD) activity and malondialdehyde (MAD) content [24]. Moreover, a study found that a novel *Bacillus*-fermented green tea could suppress pancreatic lipase activity in vitro (IC_50_ = 0.48 mg/mL) and reduce postprandial lipaemia by 26% with 500 mg/kg tea in rats [46].

EGCG, the most abundant catechin in green tea, could attenuate the endothelial dysfunction induced by oxidized-LDL through the Jagged-1/Notch signaling pathway in human umbilical vein endothelial cells. Meanwhile, in apolipoprotein E (ApoE) knockout mice, EGCG was also confirmed to be effective in alleviating high-fat diet (HFD)-induced atherosclerosis through the Jagged-1/Notch signaling pathway [47]. Furthermore, the administration of EGCG to HFD-fed ApoE^−/−^ mice significantly inhibited atherosclerotic plaque formation with upregulation of interleukin-10 (IL-10) levels and downregulation of plasma IL-6 and tumor necrosis factor-α (TNF-α) levels and attenuated HFD-induced dyslipidemia through modulating the liver X receptor (LXR)/sterol regulatory element binding transcription factor-1(SREBP-1) pathway [48]. In addition, green tea catechins were found to be effective in inhibiting LDL oxidation through incorporating themselves into LDL particles in nonconjugated forms in vitro [49]. Epicatechin could lower TC, LDL-C, and TG, mitigate liver fat accumulation, and increase HDL-C in hyperlipidemic rats induced by high-fat, high-cholesterol diets. These alterations were achieved through regulating the Insig-1-SREBP-SCAP pathway and other lipid metabolic-associated genes, including LXR-α, fatty acid synthase (FAS) and sirtuin 1 (SIRT1) [50]. Furthermore, a study revealed that green tea polyphenol could improve lipid metabolism disorders, inhibit atherogenesis, and elevate the expression of hepatic PPAR α and autophagy markers (LC3, Beclin1, and p62) in the vessel wall of ApoE-knockout mice [51].

### 3.2. Ameliorating Ischemia/Reperfusion Injury

Studies on ischemia/reperfusion have indicated the protective role of tea extract against ischemia/reperfusion related injuries [52,53]. A polyphenol trimer from green tea, cinnamon, and resveratrol were found effective in decreasing mitochondrial reactive oxygen species (ROS) and cell swelling in endothelial cells suffering from ischemic injury [52]. In addition, green tea showed a stronger effect than other teas against ischemia/reperfusion in male Wistar rats [53]. In another study, EGCG in combination with zinc could inhibit hypoxia/reoxygenation-induced cell apoptosis through activating the phosphatidylinositol-3-kinase (PI3K)/RAC-α serine/threonine-protein kinase (Akt) signaling pathway in H9c2 rat cardiac myoblast cells [54]. Additionally, pretreatment of EGCG to H9c2 cells could reduce the apoptosis induced by hypoxia/reoxygenation through stabilizing mitochondrial membrane potential and decreasing the expression of mitochondrial damage-related proteins [55]. Pretreatment of EGCG to albino Westar rats could protect against myocardial infarction induced by isoproterenol through reducing myocardial apoptosis. The related cardio-protective effects of EGCG was achieved by sustaining the balance of anti-apoptotic/pro-apoptotic proteins involved in the mitochondrial apoptotic pathway, restricting oxidative stress, and maintaining the integrity of DNA [56]. Besides, EGCG post-conditioning could attenuate ischemia/reperfusion injury and inhibit myocardial apoptosis via the PI3K/Akt signaling pathway in rats [57]. Additionally, EGCG showed cardioprotective effects, including alleviating myocardial injury and preventing ventricular arrhythmia in a rat ischemia/reperfusion model, with the mechanisms inhibiting the release of mitochondrial DNA (a potent pro-inflammatory mediator) and regulating the PI3K/Akt signaling pathway [58]. EGCG could improve hemodynamic recovery during reperfusion, elevate the adenosine triphosphate (ATP)-level, and relieve oxidative stress in excised perfused rabbit hearts [59]. Epicatechin could protect from cardiac injury induced by ischemia and inhibit myocardial apoptosis, cardiac fibrosis, and myocardial hypertrophy, which was achieved by the phosphatase and tensin homolog (PTEN)/PI3K/Akt signaling pathway [60]. Catechin could alleviate hypoxia-induced injuries through decreasing microRNA-92a and modulating the JNK signaling pathway in H9c2 cells [61].

### 3.3. Protecting Endothelial Function

Many studies have revealed that tea and its bioactive compounds could improve endothelial function. Black tea administration, rich in theaflavins, could prevent endothelial dysfunction, reduce the reduced form of nicotinamide adenine dinucleotide phosphate (NADPH) oxidase, serum total cholesterol and ROS levels, and restore the level of phospho endothelial nitric oxide synthase (eNOS) in ovariectomized rats [62]. Another study reported that black tea could alleviate the endothelial injury caused by hypertension via reducing the serum homocysteine level and endothelial cell endoplasmic reticulum stress in male Sprague Dawley rats [63]. Hyperhomocysteinemia would cause vascular endothelial dysfunction and promote the development of atherosclerosis. EGCG was effective in inhibiting homocysteine-induced apoptosis via regulating the mitochondrial apoptotic and PI3K/Akt/eNOS signaling pathways in human umbilical vein endothelial cells [64]. Besides, EGCG could inhibit the proliferation of vascular smooth muscle cell induced by homocysteine with ERK1/2 and p38MAPK signaling pathways involved [65]. Moreover, EGCG could stimulate the proliferation, migration, and tube formation of endothelial cells and promote angiogenesis in mice through transient receptor potential vanilloid type 1(TRPV1) activation [66].

### 3.4. Protecting Cardiomyocyte Function

Oolong tea could dose-dependently alleviate 24 h hypoxia-induced cardiomyocyte loss and hypertrophy through inhibiting caspase-3-cleavage and apoptosis and enhancing *p*-Akt-associated survival [67]. Additionally, EGCG could normalize the increased Ca^2+^ sensitivity of myofilaments caused by a mutation in human cardiac troponin i (k206i), which is related to hypertrophic cardiomyopathy [68]. Moreover, EGCG was found to be effective in reducing cardiac hypertrophy and fibrosis through increasing the diameter and volume of cardiomyocytes and decreasing the generation of ROS in aged rats [69]. Furthermore, EGCG could protect the heart development of zebrafish embryos from injuries caused by bisphenol A, an emerging contaminant associated with CVDs [70].

### 3.5. Reducing Oxidative Stress

Oxidative stress is closely associated with many chronic diseases, such as cardiovascular diseases [71]. Green tea and γ-amino butyric acid (GABA) green tea are rich in polyphenol, theanine, glutamine, and caffeine, which were found to be effective in reducing oxidative stress, modulating antioxidant endogenous defenses, and improving post-stroke depression in mice [71]. Besides, a study reported that treatment of white tea could improve cardiac glycolytic and heart antioxidant capacity in prediabetic rats [72]. Moreover, EGCG could prevent human umbilical vein endothelial cells from oxidative stress injury induced by PM_2.5_, an ambient fine particulate matter which could cause certain CVDs. These antioxidant effects of EGCG were achieved by activating the p38 mitogen-activated protein kinase (MAPK) and extracellular signal regulated kinase (ERK)1/2 signaling pathways and subsequently upregulating the nuclear factor E2-related factor 2 (Nrf2)/heme oxygenase-1 (HO-1) pathway [73]. Furthermore, EGCG could decrease myocardial oxidative stress and free fatty-acid levels, thus inhibiting the development of heart failure induced by the heart/muscle-specific deletion of manganese superoxide dismutase in mice [74]. Theanine could protect H9c2 cells against hydrogen peroxide-induced apoptosis via enhancing antioxidant capacity, such as elevating the activities of glutathione peroxidase and SOD, and reducing the levels of ROS, nitric oxide, and oxidized glutathione [75].

### 3.6. Alleviating Inflammation

Inflammation is involved in the development of many metabolic diseases, such as CVDs, obesity, and cancers [76]. A study reported that EGCG could suppress the production of blood angiotensin II-associated C-reactive protein, which plays a vital role in the progression of atherosclerosis and inflammatory hepatic diseases, through the angiotensin II type 1 receptor-ROS-ERK1/2 signaling pathway [77]. EGCG could also inhibit the inflammatory response via regulating the Notch pathway in human macrophages [78]. Moreover, EGCG alleviated inflammation through the increase of E3 ubiquitin ligase RNF 216, followed by downregulation of toll-like receptor 4 [79]. Additionally, green tea extract treatment could attenuate cardiac macrophage infiltration and improve insulin secretion function through activating the adenosine monophosphate-activated protein kinase (AMPK) signaling pathway in weanling rats [80].

It’s noteworthy that the bioavailability of tea polyphenols is usually very poor, for example, the peak plasma concentration of EGCG was only 0.15 μM after consuming two cups of green tea in humans [81,82]. Tea catechins are deeply modified by gut bacteria. Phenyl-γ-valerolactones and phenylvaleric acids are the major gut metabolic products of catechins and have been found to be effective in preventing chronic diseases [83]. Besides, black tea theaflavin and its galloyl derivatives are barely absorbed in human digestive tracts, but the galloyl part of theaflavin released by the microbiota has been reported to have many bioactivities [84]. Moreover, it has been reported that black tea has similar effects to green tea in protecting cardiovascular diseases [9]. Although green tea is more abundant in catechins than black tea, black tea is rich in theaflavins and thearubigins, which could compensate its functions due to the lack of catechins.

**Figure 2:**
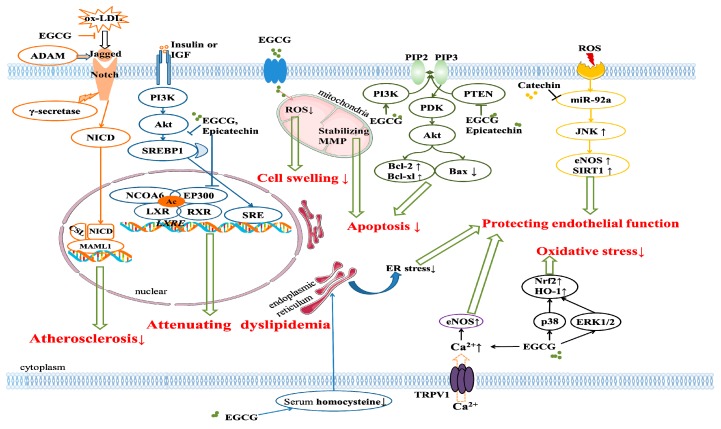
Signaling pathways involved in the protective effects of tea bioactive compounds against cardiovascular diseases. Epigallocatechin-3-gallate (EGCG) reduced atherosclerosis by inhibiting the activation of the Notch receptor induced by oxidized-LDL. EGCG and epicatechin could attenuate dyslipidemia through regulating the SREBP1 pathway. EGCG could reduce the reactive oxygen species level in mitochondria and stabilize the mitochondrial membrane potential, thus attenuating cell swelling and apoptosis of endothelial cells. EGCG and epicatechin could reduce the apoptosis of cardiac cells through regulating the PI3K pathway. EGCG could protect endothelial function through alleviating endoplasmic reticulum stress. EGCG and catechin could elevate the endothelial nitric oxide synthase (eNOS), thus protecting endothelial function. EGCG could reduce oxidative stress by regulating the p38 MAPK and ERK1/2 pathways. Abbreviations: ADAM, A-Disintegrin-And-Metalloprotease; NICD, Notch intracellular domain; PI3K, phosphatidylinositol-3-kinase; Akt, α serine/threonine-protein kinase; SREBP, sterol regulatory element binding transcription factor; LXR, liver X receptor; RXR, retinoid X receptor; NCOA6, nuclear receptor coactivator 6; PTEN, phosphatase and tensin homolog; PDK, phosphoinositide dependent kinase; Nrf, nuclear factor E2-related factor; HO-1, heme oxygenase-1; TRPV, transient receptor potential vanilloid type.

Collectively, tea, especially green tea, black tea, and white tea, and their bioactive compounds, such as EGCG, catechin, and theanine, possess remarkable protective effects against CVDs. The effects of tea on CVDs by in vitro and in vivo experimental studies are summarized in Table 2. The main cardiovascular protective mechanisms of tea include the reduction of blood lipid, alleviation of ischemia/reperfusion injury, enhancement of endothelial function, protection of cardiomyocytes, attenuation of oxidative stress, and relief of inflammation (Figure 2).

## 4. Clinical Trails

Dyslipidemia is a major risk factor of CVD development [43]. Benifuuki green tea, containing O-methylated catechin, was shown to remarkably reduce serum concentrations of LDL-C and lectin-like oxidized low-density lipoprotein receptor-1 ligands containing apolipoprotein B (LAB) in a randomized controlled trial (RCT) conducted on 155 volunteers [85]. Another RCT including 151 volunteer subjects also showed that Benifuuki and Yabukita green teas could decrease the LDL-C level [86]. In addition, consumption of green tea catechin extract for one year could lower serum TC (*p* = 0.0004), LDL-C (*p* < 0.0001), and non-HDL cholesterol (*p* = 0.0032) in healthy postmenopausal women [26]. A study investigated the hypolipidemic and antioxidant activities of catechin-enriched green and oolong teas in mild hypercholesterolemic individuals and found that the two teas showed similar antioxidant capacity and catechin-enriched oolong tea exerted a stronger hypolipidemic activity [87]. In addition, a meta-analysis concerned with ten RCTs of black tea drinking and serum cholesterol level found that black tea greatly reduced the serum LDL-C level, especially in those with higher risk of CVDs [25]. In another RCT involving 99 participants aged 25–60 years old with mild hypercholesterolemia, phytosterol-enriched instant black tea was found to be effective in lowering the levels of total cholesterol (*p* < 0.001), LDL-C (*p* < 0.001), apolipoprotein B (*p* < 0.05), and oxidative stress (*p* < 0.05), and elevating the levels of adiponectin, total antioxidant capacity, and tissue-plasminogen activator (*p* < 0.05), which were beneficial to cardiovascular function [88].

Hypertension is an independent predictor of cardiovascular-related death [89]. A study investigated the antihypertensive effects of short-term green tea consumption in 15 young volunteers aged 18–35 years old and 15 older volunteers aged 55–75 years old, and found that green tea could improve SBP, skin microvascular function, and oxygen tension [89]. In another study, the antihypertensive property of short-term consumption of green tea was revealed by a crossover RCT in 20 obese and prehypertensive women aged 41.1 ± 8.4 years old. Compared with a placebo group, women who had been drinking green tea for four weeks showed a decrease (*p* < 0.05) in 24-hour SBP (−3.61 ± −1.23 mmHg), daytime SBP (−3.61 ± −1.26 mmHg), and nighttime SBP (−3.94 ± −1.70 mmHg), with no significant changes in DBP [90]. Moreover, dietary flavonoids obtained from green tea, dehydrated red apple, and dark chocolate, at a dose of 425.8 ± 13.9 mg epicatechin equivalents combining with antihypertensive treatments (telmisartan or captopril), were found to significantly lower SBP and DBP in a RCT with 79 hypertension patients aged 20–55 years old [91]. Furthermore, in a RCT conducted on 19 hypertensive patients, consumption of black tea for eight days was shown to reduce SBP by 3.2 mmHg (*p* < 0.005) and DBP by 2.6 mmHg (*p* < 0.0001), inhibit the increase of BP within a fat load (*p* < 0.0001), and lower the index of reflection and stiffness [92].

Black tea also showed remarkable endothelial protective effects in a RCT conducted on 19 patients with hypertension. The participants of this study consumed black tea (containing 150 mg polyphenols) or a placebo twice a day for eight days, and the results indicated that black tea could stimulate the circulating amount of angiogenic cells and improve acute oral fat load-induced dysfunction of endothelial cells [93]. Additionally, a RCT involving 50 healthy men compared the endothelial protective effects of EGCG in three formulas including a green tea beverage, green tea extract, and pure EGCG, and found that only the green tea beverage could improve flow-mediated dilation [94]. Another RCT conducted with 14 healthy participants found that the intake of green tea polyphenol-enriched ice cream could immediately enhance vascular function and reduce oxidative stress [95]. Moreover, the administration of epicatechin (100 mg/d) for four weeks was found to improve the endothelial function and attenuate inflammation in 37 (pre)hypertensive participants [96].

In summary, numerous clinical trials (Table 3) support the cardiovascular-protective properties of tea and its bioactive compounds, with main mechanisms including reducing blood lipids, lowering BP, and protecting endothelial function.

## 5. Conclusions

Results from substantial epidemiological research has indicated that tea consumption is reversely associated with CVD risks, especially in those drinking tea habitually. In addition, a number of in vitro and in vivo experimental studies supported the protective effects of tea and its bioactive compounds against CVDs. The underlying mechanisms of action mainly include reducing blood lipid, alleviating ischemia/reperfusion injury, protecting cardiomyocyte function, enhancing endothelial function, lowering oxidative stress, and attenuating inflammation. Furthermore, clinical trials have also revealed that tea consumption could protect against CVDs. Therefore, it is valuable to recommend tea consumption for the public to protect cardiovascular health. Except for catechins and theaflavins, tea also contained procyanidin, phenolic acids, and so on. For example, the total proanthocyanidins in nine Mauritian black teas varied from 25 ± 2 to 74 ± 10 mg cyanidin chloride/g dry weight [97]. But few studies have focused on the effects of tea procyanidin and phenolic acids against cardiovascular diseases. In the future, different teas should be further evaluated considering their cardiovascular protective effects from the bench to bed, and more effective compounds should be separated and identified. More importantly, the mechanisms of action of teas, such as the molecular targets and mediated signaling pathways, should be further clarified to provide a better understanding for the action of tea. Last but not least, the safety of tea should be paid attention to, since its health benefits must be established on its safety.

## Figures and Tables

**Table 1:** The effects of tea on CVDs based on epidemiological studies.

Subjects	Study Type	Effects	Risk Estimates (95%CI)	Ref.
90,914 Japanese participants aged 40–69 y	cohort study	Reducing the risk of heart disease and cerebrovascular disease	heart disease: 0.70 (0.56–0.87) for 3–4 cups/day; cerebrovascular disease: 0.73 (0.56–0.94) for 3–4 cups/day	[27]
165,000 Chinese adult men without pre-existing disease	cohort study	Reducing the risk of CVDs	0.93(0.85–1.01) for ≤5 g/day; 0.91 (0.85–0.98) for 5–10 g/day; 0.86 (0.79–0.93) for >10 g/day	[22]
74,941 women aged 40–70 y and 61,491 men aged 40–74 y in China	cohort study	Reducing the risk of CVDs	0.86 (0.77–0.97)	[28]
120,852 men and women in the Netherlands aged 55–69 y	cohort study	Reducing the risk of CVDs	0.72 (0.57–0.91) for 2–3 cups/day in men	[29]
19,471 participants free of CHD, stroke or cancer	cohort study	Reducing the risk of CHD	0.89 (0.81–0.98)	[30]
6508 participants from Multi-Ethnic Study of Atherosclerosis	cohort study	Slowing the progression of coronary artery calcium	0.71 (0.53–0.95) for ≥1 cup/day	[31]
1063 women aged >75 y in Australia	cohort study	Reducing the mortality of CVDs	0.34 (0.17–0.69) for data from USDA; 0.32 (0.16–0.61) for data from Phenol-Explorer databases	[32]
774 Dutch men aged 65–84 y	cohort study	Reducing the risk of CVDs	0.54 (0.31–0.96)	[23]
80,182 Chinese participants aged 37–61 y free of CVDs, cancers, and cholesterol-lowering agent use	cohort study	Increasing blood HDL-C	NA	[40]
29,876 participants aged 40–69 y free of heart disease, stroke, or cancer in Japan	case-control study	Lowering the risk of stroke in non-smoking men	0.53 (0.29–0.98)	[36]
1352 participants aged 18–69 y in Luxembourg	cross-sectional study	Decreasing the SBP and pulse pressure	NA	[38]
4579 participants aged ≥60 y in China	cross-sectional study	Lowering DBP and the risk of hypertension	0.79 (0.65–0.95)	[39]
5856 participants (case 2909, control 2947) in China	case-control study	Increasing the risk of acute myocardial infarction	1.29 (1.03–1.61) for 4 cups/d	[42]

Abbreviations: y, year; NA, Not available; CVD, cardiovascular disease; CHD, coronary heart disease; SBP, systolic blood pressure; DBP, diastolic blood pressure.

**Table 2:** The effects and mechanisms of tea on CVDs based on in vitro and in vivo experimental studies.

Substances	Subjects	Study Type	Dose	Effects and Mechanisms	Ref.
Green tea extract	Male C57BL/6J mice	In vivo	0.2% (*w*/*w*)	HMGCR↓, HMGCS↓, cholesterol↓	[45]
Matcha	Male ICR mice	In vivo	0.025%, 0.05%, 0.075% (*w*/*w*)	TC↓, TG↓, LDL-C↓, serum glucose↓; HDL↑, SOD↑, MAD↑	[24]
*Bacillus*-fermented green tea	Pancreatic lipase; Sprague-Dawley male Rats	In vitro and in vivo	IC_50_ 0.48 mg/mL; 500mg/kg	TG↓, pancreatic lipase activity↓	[46]
Green tea infusion	Male Wistar rats	In vivo	400 mg/kg	Hippocampal oxidative stress↓, necrosis↓	[53]
GABA green tea	Male balb/c mice	In vivo	50 and 100 mg/kg	Oxidative stress↓; Antioxidant endogenous defenses↑	[71]
Green tea extract	Pregnant Wistar rats	In vivo	0.12%, 0.24%	Cardiac macrophage infiltration↓; Insulin↑	[80]
Black tea	Female Sprague-Dawley rats	In vivo	15 mg/kg/day	NADPH oxidases↓, ROS↓; Flow-mediated dilatation↑	[62]
Black tea	Rat aortic endothelial cells; Male Sprague Dawley rats	In vitro and in vivo	0.3–5 μM; 15 mg/kg/day	Endothelial injury↓, serum homocysteine↓, endoplasmic reticulum stress↓	[63]
Oolong tea	H9c2 cardiac myoblast cells; Neonatal rat ventricular cardiomyocytes	In vitro	100, 200, 400 mg/mL	Cardiomyocyte loss ↓, hypertrophy ↓	[67]
White tea	Male Wistar rats	In vivo	1 g/100 mL	Cardiac glycolytic↑, antioxidant capacity↑	[72]
EGCG	Human umbilical vein endothelial cells; ApoE^−/−^ mice	In vitro and in vivo	50 µM; 0.8 g/L	Endothelial dysfunction↓; Jagged-1/Notch activated	[47]
EGCG	ApoE^−/−^ mice	In vivo	40 mg/kg/d	IL-6↓, TNF-α↓, TG↓, TC↓, LDL↓; IL-10↑, HDL↑, LXR/SREBP-1 pathways modulated	[48]
EGCG	H9c2 cardiac myoblast cells	In vitro	5, 10, 15, and 20 µM	Hypoxia/reoxygenation induced apoptosis↓	[54]
EGCG	H9c2 cardiac myoblast cells	In vitro	10 μM	Apoptosis↓; Stabilizing mitochondrial membrane potential	[55]
EGCG	Albino Westar rats	In vivo	15 mg/kg.	Myocardial infarction↓	[56]
EGCG	Male Sprague-Dawley rats	In vivo	10 mg/kg	Myocardial apoptosis↓	[57]
EGCG	Chinchilla rabbit heart	In vitro	20 μM/L	Oxidative stress↓; ATP↑	[59]
EGCG	Male Wistar rats	In vivo	10 mg/kg	Plasma mtDNA↓, TNF↓, IL-6 ↓, IL-8↓, ventricular arrhythmia↓	[58]
EGCG	Human umbilical vein endothelial cells	In vitro	10, 20, 30 µM	Apoptosis↓	[64]
EGCG	Human aortic smooth muscle cells	In vitro	20 µM	Homocysteine-induced proliferation↓	[65]
EGCG	Bovine aortic endothelial cells; WT C57BL mice and TRPV1^−/−^ mice	In vitro and in vivo	0, 1.25, 2.5, 10, 20 μM; 10 μM	Angiogenesis↑	[66]
EGCG	Wistar albino rats	In vivo	200 mg/kg	Cardiac hypertrophy↓, fibrosis↓, LDL↓, VLDL↓, TG↓, TC↓; HDL↑, TGFβ↑, TNFα↑, NF-κB↑	[69]
EGCG	Zebrafish embryos	In vivo	50, 100 μM	Damage caused by bisphenol A↓	[70]
EGCG	Human umbilical vein endothelial cells	In vitro	50, 100, 200, 300, 400 μM	Oxidative stress↓; Nrf2↑, HO-1↑	[73]
EGCG	Heart/muscle-specific MnSOD-deficient mice	In vivo	10 mg/L, 100 mg/L	Myocardial oxidative stress↓, free fatty acid↓	[74]
EGCG	Male Sprague-Dawley rats	In vivo	25, 50 mg/kg/day	Ang II type 1 receptor↓, ERK1/2↓; PPARγ ↑	[77]
EGCG	Human monocyte cell line	In vitro	50 µg/mL	Inflammatory response↓	[78]
EGCG	Male C57/BL6 mice	In vivo	0, 2.5, 5, 10 μM	TLR4 expression↓	[79]
Epicatechin	Male Sprague-Dawley rats	In vivo	10, 20, 40 mg/kg	TC↓, LDL-C↓, TG↓; HDL-C↑	[50]
Theanine	H9c2 cardiac myoblast cells	In vitro	0, 4, 8, 16 μM	Peroxide-induced apoptosis↓, ROS↓; SOD↑	[75]

Up arrows mean increase, down arrows mean decrease. HMGCR, 3-hydroxy-3-methylglutary-coenzyme A reductase; HMGCS, 3-hydroxy-3-methylglutary-coenzyme A synthase; TC, total cholesterol; TG, triglyceride; LDL-C, low-density lipoprotein-cholesterol; HDL, high-density lipoprotein; SOD, superoxide dismutase; MAD, malondialdehyde; NADPH, nicotinamide adenine dinucleotide phosphate; ROS, reactive oxygen species; IL, interleukin; TNF, tumor necrosis factor; VLDL, very low-density lipoprotein; TGF, transforming growth factor; NF, nuclear factor; Nrf2, nuclear factor E2-related factor 2; HO-1, heme oxygenase-1; ERK, extracellular signal-regulated kinases; PPAR, peroxisome proliferator-activated receptor; TLR, toll-like receptor.

**Table 3:** The effects of tea against CVDs based on clinical studies.

Subjects	Substances	Treatments	Effects and Mechanisms	Ref.
155 healthy participants	A green tea containing *O*-methylated catechin	12 g/d for 12 weeks	LDL-C↓, LAB↓	[85]
151 participants aged 30–70 y	Green tea	1.8 g/d for 12 weeks	LDL-C↓	[86]
15 participants aged 18–35 y and 15 participants aged 55–75 y	Green tea	2 cups/d for 14 days	Improving SBP and skin microvascular function	[89]
20 women aged 32.7–49.5 y	Green tea extract	500 mg for 4 weeks	SBP↓	[90]
50 healthy men	Green tea	A single dose of 200 mg EGCG	Improving flow-mediated dilation	[94]
14 healthy individuals	Green tea polyphenol-enriched ice cream	A single dose of 100 g	Oxidative stress↓,Vascular function↑	[95]
79 hypertension patients aged 20–55 y	Flavonoids from green tea	425.8 ± 13.9 mg epicatechin equivalents for 6 months	SBP ↓, DBP↓	[91]
60 individuals with mild hypercholesterolemia	Catechin-enriched green or oolong tea	780.6 mg/d or 640.4 mg/d catechin for 12 weeks	TC↓, LDL-C↓, TG↓	[87]
1075 healthy postmenopausal women	Catechins	1315 mg for 1 year	TC↓, LDL-C↓, non-HDL-C levels↓	[26]
99 participants aged 25–60 y with mild hypercholesterolemia	Phytosterol-enriched instant black tea	2.5 g/d for 4 weeks	Blood lipids↓	[88]
19 hypertensive patients	Black tea	129 mg/d flavonoids for 8 days	SBP↓	[92]
19 hypertension patients	Black tea	150 mg polyphenols for 8 days	Endothelial function↑	[93]
37 (Pre)hypertensive participants aged 40–80 y	Epicatechin or quercetin-3-glucoside	100 mg/d or 160 mg/d, respectively, for 4 weeks	Inflammation↓,Endothelial function ↑	[96]

Up arrows mean increase, down arrows mean decrease; LDL-C, low-density lipoprotein-cholesterol; LAB, apolipoprotein B; SBP, systolic blood pressure; DBP, diastolic blood pressure; TC, total cholesterol; TG, triglyceride; HDL, high-density lipoprotein.

## References

[1] Cardiovascular Disease. https://www.who.int/cardiovascular_diseases/en/.

[2] Yusuf S., Reddy S., Ounpuu S., Anand S. (2001). Global burden of cardiovascular diseases: part I: general considerations, the epidemiologic transition, risk factors, and impact of urbanization. Circulation.

[3] Zhao C.N., Meng X., Li Y., Li S., Liu Q., Tang G.Y., Li H.B. (2017). Fruits for prevention and treatment of cardiovascular diseases. Nutrients.

[4] Tang G.Y., Meng X., Li Y., Zhao C.N., Liu Q., Li H.B. (2017). Effects of vegetables on cardiovascular diseases and related mechanisms. Nutrients.

[5] Zheng J., Zhou Y., Li S., Zhang P., Zhou T., Xu D.P., Li H.B. (2017). Effects and mechanisms of fruit and vegetable juices on cardiovascular diseases. Int. J. Mol. Sci..

[6] Deng G.F., Xu X.R., Guo Y.J., Xia E.Q., Li S., Wu S., Chen F., Ling W.H., Li H.B. (2012). Determination of antioxidant property and their lipophilic and hydrophilic phenolic contents in cereal grains. J. Funct. Foods.

[7] Zheng J., Zhou Y., Li Y., Xu D.P., Li S., Li H.B. (2016). Spices for prevention and treatment of cancers. Nutrients.

[8] Guo Y.J., Deng G.F., Xu X.R., Wu S., Li S., Xia E.Q., Li F., Chen F., Ling W.H., Li H.B. (2012). Antioxidant capacities, phenolic compounds and polysaccharide contents of 49 edible macro-fungi. Food Funct..

[9] Angelino D., Godos J., Ghelfi F., Tieri M., Titta L., Lafranconi A., Marventano S., Alonzo E., Gambera A., Sciacca S. (2019). Fruit and vegetable consumption and health outcomes: an umbrella review of observational studies. Int. J. Food Sci. Nutr..

[10] Aune D., Keum N., Giovannucci E., Fadnes L.T., Boffetta P., Greenwood D.C., Tonstad S., Vatten L.J., Riboli E., Norat T. (2016). Whole grain consumption and risk of cardiovascular disease, cancer, and all cause and cause specific mortality: Systematic review and dose-response meta-analysis of prospective studies. BMJ.

[11] Kim Y., Keogh J., Clifton P.M. (2018). Nuts and cardio-metabolic disease: A review of meta-analyses. Nutrients.

[12] Pang J., Zhang Z., Zheng T.Z., Bassig B.A., Mao C., Liu X., Zhu Y., Shi K., Ge J., Yang Y.J. (2016). Green tea consumption and risk of cardiovascular and ischemic related diseases: A meta-analysis. Int. J. Cardiol..

[13] Grosso G., Stepaniak U., Micek A., Topor-Mądry R., Pikhart H., Szafraniec K., Pająk A. (2015). Association of daily coffee and tea consumption and metabolic syndrome: results from the Polish arm of the HAPIEE study. Eur. J. Nutr..

[14] Hodgson J.M., Croft K.D. (2010). Tea flavonoids and cardiovascular health. Mol. Aspects Med..

[15] Higdon J.V., Frei B. (2003). Tea catechins and polyphenols: Health effects, metabolism, and antioxidant functions. Crit. Rev. Food Sci. Nutr..

[16] Li S., Gan L.Q., Li S.K., Zheng J.C., Xu D.P., Li H.B. (2014). Effects of herbal infusions, tea and carbonated beverages on alcohol dehydrogenase and aldehyde dehydrogenase activity. Food Funct..

[17] Li F., Li S., Li H.B., Deng G.F., Ling W.H., Xu X.R. (2013). Antiproliferative activities of tea and herbal infusions. Food Funct..

[18] Xu X.Y., Zhao C.N., Cao S.Y., Tang G.Y., Gan R.Y., Li H.B. (2019). Effects and mechanisms of tea for the prevention and management of cancers: An updated review. Crit. Rev. Food Sci. Nutr..

[19] Wang X., Ouyang Y.Y., Liu J., Zhao G. (2014). Flavonoid intake and risk of CVD: A systematic review and meta-analysis of prospective cohort studies. Br. J. Nutr..

[20] Grosso G., Micek A., Godos J., Pajak A., Sciacca S., Galvano F., Giovannucci E.L. (2017). Dietary flavonoid and lignan intake and mortality in prospective cohort studies: systematic review and dose-response meta-analysis. Am. J. Epidemiol..

[21] Grosso G., Godos J., Lamuela-Raventos R., Ray S., Micek A., Pajak A., Sciacca S., D’Orazio N., Del Rio D., Galvano F. (2017). A comprehensive meta-analysis on dietary flavonoid and lignan intake and cancer risk: Level of evidence and limitations. Mol. Nutr. Food Res..

[22] Liu J.X., Liu S.W., Zhou H.M., Hanson T., Yang L., Chen Z.M., Zhou M.G. (2016). Association of green tea consumption with mortality from all-cause, cardiovascular disease and cancer in a Chinese cohort of 165,000 adult men. Eur. J. Epidemiol..

[23] Dower J.I., Geleijnse J.M., Hollman P.C.H., Soedamah-Muthu S.S., Kromhout D. (2016). Dietary epicatechin intake and 25-y risk of cardiovascular mortality: The Zutphen Elderly Study. Am. J. Clin. Nutr..

[24] Xu P., Ying L., Hong G.J., Wang Y.F. (2016). The effects of the aqueous extract and residue of Matcha on the antioxidant status and lipid and glucose levels in mice fed a high-fat diet. Food Funct..

[25] Zhao Y.M., Asimi S., Wu K.J., Zheng J.S., Li D. (2015). Black tea consumption and serum cholesterol concentration: Systematic review and meta-analysis of randomized controlled trials. Clin. Nutr..

[26] Samavat H., Newman A.R., Wang R., Yuan J., Wu A.H., Kurzer M.S. (2016). Effects of green tea catechin extract on serum lipids in postmenopausal women: a randomized, placebo-controlled clinical trial. Am. J. Clin. Nutr..

[27] Saito E., Inoue M., Sawada N., Shimazu T., Yamaji T., Iwasaki M., Sasazuki S., Noda M., Iso H., Tsugane S. (2015). Association of green tea consumption with mortality due to all causes and major causes of death in a Japanese population: The Japan Public Health Center-based Prospective Study (JPHC Study). Ann. Epidemiol..

[28] Zhao L.G., Li H.L., Sun J.W., Yang Y., Ma X., Shu X.O., Zheng W., Xiang Y.B. (2017). Green tea consumption and cause-specific mortality: Results from two prospective cohort studies in China. J. Epidemiol..

[29] Van den Brandt P.A. (2018). Coffee or Tea? A prospective cohort study on the associations of coffee and tea intake with overall and cause-specific mortality in men versus women. Eur. J. Epidemiol..

[30] Tian C., Huang Q., Yang L.L., Légaré S., Angileri F., Yang H.D., Li X.L., Min X.W., Zhang C., Xu C.W. (2016). Green tea consumption is associated with reduced incident CHD and improved CHD-related biomarkers in the Dongfeng-Tongji cohort. Sci. Rep..

[31] Miller P.E., Zhao D., Frazier-Wood A.C., Michos E.D., Averill M., Sandfort V., Burke G.L., Polak J.F., Lima J.A.C., Post W.S. (2017). Associations of coffee, tea, and caffeine intake with coronary artery calcification and cardiovascular events. Am. J. Med..

[32] Ivey K.L., Hodgson J.M., Croft K.D., Lewis J.R., Prince R.L. (2015). Flavonoid intake and all-cause mortality. Am. J. Clin. Nutr..

[33] Grosso G., Stepaniak U., Topor-Mądry R., Szafraniec K., Pająk A. (2014). Estimated dietary intake and major food sources of polyphenols in the Polish arm of the HAPIEE study. Nutrition.

[34] Micek A., Grosso G., Polak M., Kozakiewicz K., Tykarski A., Puch Walczak A., Drygas W., Kwaśniewska M., Pająk A., On behalf of WOBASZ II Investigators (2018). Association between tea and coffee consumption and prevalence of metabolic syndrome in Poland—Results from the WOBASZ II study (2013–2014). Int. J. Food Sci. Nutr..

[35] Adriouch S., Lampuré A., Nechba A., Baudry J., Assmann K., Kesse-Guyot E., Hercberg S., Scalbert A., Touvier M., Fezeu L.K. (2018). Prospective association between total and specific dietary polyphenol intakes and cardiovascular disease risk in the Nutrinet-Santé French cohort. Nutrients.

[36] Ikeda A., Iso H., Yamagishi K., Iwasaki M., Yamaji T., Miura T., Sawada N., Inoue M., Tsugane S. (2018). Plasma tea catechins and risk of cardiovascular disease in middle-aged Japanese subjects: The JPHC study. Atherosclerosis.

[37] Sowers J.R., Epstein M., Frohlich E.D. (2001). Diabetes, hypertension, and cardiovascular disease—An update. Hypertension.

[38] Alkerwi A., Sauvageot N., Crichton G.E., Elias M.F. (2015). Tea, but not coffee consumption, is associated with components of arterial pressure. The observation of cardiovascular risk factors study in Luxembourg. Nutr. Res..

[39] Yin J.Y., Duan S.Y., Liu F.C., Yao Q.K., Tu S., Xu Y., Pan C.W. (2017). Blood pressure is associated with tea consumption: A cross-sectional study in a rural, elderly population of Jiangsu China. J. Nutr. Health Aging.

[40] Huang S.E., Li J.J., Wu Y.T., Ranjbar S., Xing A.J., Zhao H.Y., Wang Y.X., Shearer G.C., Bao L., Lichtenstein A.H. (2018). Tea consumption and longitudinal change in high-density lipoprotein cholesterol concentration in Chinese adults. J. Am. Heart Assoc..

[41] Chapman M.J., Ginsberg H.N., Amarenco P., Andreotti F., Boren J., Catapano A.L., Descamps O.S., Fisher E., Kovanen P.T., Kuivenhoven J.A. (2011). Triglyceride-rich lipoproteins and high-density lipoprotein cholesterol in patients at high risk of cardiovascular disease: Evidence and guidance for management. Eur. Heart J..

[42] Hao G., Li W., Teo K., Wang X.Y., Yang J.G., Wang Y., Liu L.S., Yusuf S. (2015). Influence of tea consumption on acute myocardial infarction in China population: The INTERHEART China Study. Angiology.

[43] Bertolotti M., Maurantonio M., Gabbi C., Anzivino C., Carulli N. (2005). Review article: hyperlipidaemia and cardiovascular risk. Aliment Pharm. Ther..

[44] Chang C., Hsu Y., Chen Y.M., Huang W.C., Huang C.C., Hsu M.C. (2015). Effects of combined extract of cocoa, coffee, green tea and garcinia on lipid profiles, glycaemic markers and inflammatory responses in hamsters. BMC Complement. Altern. Med..

[45] Yamashita M., Kumazoe M., Nakamura Y., Won Y.S., Bae J., Yamashita S., Tachibana H. (2016). The combination of green tea extract and eriodictyol inhibited high-fat/high-sucrose diet-induced cholesterol upregulation is accompanied by suppression of cholesterol synthesis enzymes. J. Nutr. Sci. Vitaminol..

[46] Seo D.B., Jeong H.W., Kim Y.J., Kim S., Kim J., Lee J.H., Joo K., Choi J.K., Shin S.S., Lee S.J. (2017). Fermented green tea extract exhibits hypolipidaemic effects through the inhibition of pancreatic lipase and promotion of energy expenditure. Brit. J. Nutr..

[47] Yin J., Huang F., Yi Y., Yin L., Peng D. (2016). EGCG attenuates atherosclerosis through the Jagged-1/Notch pathway. Int. J. Mol. Med..

[48] Pan L.L., Wu Y., Wang R.Q., Chen J.W., Chen J., Zhang Y., Chen Y., Geng M., Xu Z.D., Dai M. (2018). (−)-Epigallocatechin-3-Gallate ameliorates atherosclerosis and modulates hepatic lipid metabolic gene expression in apolipoprotein E knockout mice: Involvement of TTC39B. Front. Pharmacol..

[49] Suzuki-Sugihara N., Kishimoto Y., Saita E., Taguchi C., Kobayashi M., Ichitani M., Ukawa Y., Sagesaka Y.M., Suzuki E., Kondo K. (2016). Green tea catechins prevent low-density lipoprotein oxidation via their accumulation in low-density lipoprotein particles in humans. Nutr. Res..

[50] Cheng H., Xu N., Zhao W., Su J.J., Liang M.R., Xie Z.W., Wu X.L., Li Q.L. (2017). (−)-Epicatechin regulates blood lipids and attenuates hepatic steatosis in rats fed high-fat diet. Mol. Nutr. Food Res..

[51] Ding S.B., Jiang J.J., Yu P.X., Zhang G.F., Zhang G.H., Liu X.T. (2017). Green tea polyphenol treatment attenuates atherosclerosis in high-fat diet-fed apolipoprotein E-knockout mice via alleviating dyslipidemia and upregulating autophagy. PLoS ONE.

[52] Panickar K.S., Qin B., Anderson R.A. (2015). Ischemia-induced endothelial cell swelling and mitochondrial dysfunction are attenuated by cinnamtannin D1, green tea extract, and resveratrol in vitro. Nutr. Neurosci..

[53] Martins A., Schimidt H.L., Garcia A., Altermann C.D.C., Santos F.W., Carpes F.P., Da Silva W.C., Mello-Carpes P.B. (2017). Supplementation with different teas from *Camellia sinensis* prevents memory deficits and hippocampus oxidative stress in ischemia-reperfusion. Neurochem. Int..

[54] Zeng X., Tan X. (2015). Epigallocatechin-3-gallate and zinc provide anti-apoptotic protection against hypoxia/reoxygenation injury in H9c2 rat cardiac myoblast cells. Mol. Med. Rep..

[55] Wang W., Huang X., Shen D., Ming Z., Zheng M., Zhang J. (2018). Polyphenol epigallocatechin-3-gallate inhibits hypoxia/reoxygenation-induced H9c2 cell apoptosis. Minerva Med..

[56] Othman A.I., Elkomy M.M., El-Missiry M.A., Dardor M. (2017). Epigallocatechin-3-gallate prevents cardiac apoptosis by modulating the intrinsic apoptotic pathway in isoproterenol-induced myocardial infarction. Eur. J. Pharmacol..

[57] Xuan F., Jian J. (2016). Epigallocatechin gallate exerts protective effects against myocardial ischemia/reperfusion injury through the PI3K/Akt pathway-mediated inhibition of apoptosis and the restoration of the autophagic flux. Int. J. Mol. Med..

[58] Qin C.Y., Zhang H.W., Gu J., Xu F., Liang H.M., Fan K.J., Shen J.Y., Xiao Z.H., Zhang E.Y., Hu J. (2017). Mitochondrial DNA-induced inflammatory damage contributes to myocardial ischemia reperfusion injury in rats: Cardioprotective role of epigallocatechin. Mol. Med. Rep..

[59] Salameh A., Schuster R., Daehnert I., Seeger J., Dhein S. (2018). Epigallocatechin gallate reduces ischemia/reperfusion injury in isolated perfused rabbit hearts. Int. J. Mol. Sci..

[60] Li J.W., Wang X.Y., Zhang X., Gao L., Wang L.F., Yin X.H. (2018). (−)-Epicatechin protects against myocardial ischemia-induced cardiac injury via activation of the PTEN/PI3K/AKT pathway. Mol. Med. Rep..

[61] Fang J.F., Dai J.H., Ni M., Cai Z.Y., Liao Y.F. (2018). Catechin protects rat cardiomyocytes from hypoxia-induced injury by regulating microRNA-92a. Int. J. Clin. Exp. Pathol..

[62] Leung F.P., Yung L.M., Ngai C.Y., Cheang W.S., Tian X.Y., Lau C.W., Zhang Y., Liu J., Chen Z.Y., Bian Z.X. (2016). Chronic black tea extract consumption improves endothelial function in ovariectomized rats. Eur. J. Nutr..

[63] Cheang W.S., Ngai C.Y., Tam Y.Y., Tian X.Y., Wong W.T., Zhang Y., Lau C.W., Chen Z.Y., Bian Z.X., Huang Y. (2015). Black tea protects against hypertension-associated endothelial dysfunction through alleviation of endoplasmic reticulum stress. Sci. Rep..

[64] Liu S.M., Sun Z.W., Chu P., Li H.L., Ahsan A., Zhou Z.R., Zhang Z.H., Sun B., Wu J.J., Xi Y.L. (2017). EGCG protects against homocysteine-induced human umbilical vein endothelial cells apoptosis by modulating mitochondrial-dependent apoptotic signaling and PI3K/Akt/eNOS signaling pathways. Apoptosis.

[65] Zhan X.L., Yang X.H., Gu Y.H., Guo L.L., Jin H.M. (2018). Epigallocatechin gallate protects against homocysteine-induced vascular smooth muscle cell proliferation. Mol. Cell Biochem..

[66] Guo B.C., Wei J., Su K.H., Chiang A.N., Zhao J.F., Chen H.Y., Shyue S.K., Lee T.S. (2015). Transient receptor potential vanilloid type 1 is vital for (−)-epigallocatechin-3-gallate mediated activation of endothelial nitric oxide synthase. Mol. Nutr. Food Res..

[67] Shibu M.A., Kuo C., Chen B., Ju D., Chen R., Lai C., Huang P., Viswanadha V.P., Kuo W., Huang C. (2018). Oolong tea prevents cardiomyocyte loss against hypoxia by attenuating p-JNK mediated hypertrophy and enhancing P-IGF1R, p-Akt, and p-Bad^ser136^ activity and by fortifying Nrf2 antioxidation system. Environ. Toxicol..

[68] Warren C.M., Karam C.N., Wolska B.M., Kobayashi T., de Tombe P.P., Arteaga G.M., Bos J.M., Ackerman M.J., Solaro R.J. (2015). Green tea catechin normalizes the enhanced Ca^2+^ sensitivity of myofilaments regulated by a hypertrophic cardiomyopathy-associated mutation in human cardiac troponin I (K206I). Circ-Cardiovasc. Genet..

[69] Muhammed I., Sankar S., Govindaraj S. (2018). Ameliorative effect of epigallocatechin gallate on cardiac hypertrophy and fibrosis in aged rats. J. Cardiovasc. Pharm..

[70] Lombo M., Gonzalez-Rojo S., Fernandez-Diez C., Paz Herraez M. (2019). Cardiogenesis impairment promoted by bisphenol A exposure is successfully counteracted by epigallocatechin gallate. Environ. Pollut..

[71] Di Lorenzo A., Nabavi S.F., Sureda A., Moghaddam A.H., Khanjani S., Arcidiaco P., Nabavi S.M., Daglia M. (2016). Antidepressive-like effects and antioxidant activity of green tea and GABA green tea in a mouse model of post-stroke depression. Mol. Nutr. Food Res..

[72] Alves M.G., Martins A.D., Teixeira N.F., Rato L., Oliveira P.F., Silva B.M. (2015). White tea consumption improves cardiac glycolytic and oxidative profile of prediabetic rats. J. Funct. Foods.

[73] Yang G.Z., Wang Z.J., Bai F., Qin X.J., Cao J., Lv J.Y., Zhang M.S. (2015). Epigallocatechin-3-gallate protects HUVECs from PM_2.5_-induced oxidative stress injury by activating critical antioxidant pathways. Molecules.

[74] Oyama J.I., Shiraki A., Nishikido T., Maeda T., Komoda H., Shimizu T., Makino N., Node K. (2017). EGCG, a green tea catechin, attenuates the progression of heart failure induced by the heart/muscle-specific deletion of MnSOD in mice. J. Cardiol..

[75] Li C., Yan Q., Tang S., Xiao W., Tan Z. (2018). L-Theanine protects H9c2 cells from hydrogen peroxide-induced apoptosis by enhancing antioxidant capability. Med. Sci. Monit..

[76] Hotamisligil G.S. (2006). Inflammation and metabolic disorders. Nature.

[77] Zhao J., Liu J., Pang X., Zhang X., Wang S., Wu D. (2016). Epigallocatechin-3-gallate inhibits angiotensin II-induced C-reactive protein generation through interfering with the AT_1_-ROS-ERK1/2 signaling pathway in hepatocytes. Naunyn Schmiedebergs Arch. Pharmacol..

[78] Wang T.F., Xiang Z.M., Wang Y., Li X., Fang C.Y., Song S., Li C.L., Yu H.S., Wang H., Yan L. (2017). (−)-Epigallocatechin gallate targets Notch to attenuate the inflammatory response in the immediate early stage in human macrophages. Front. Immunol..

[79] Kumazoe M., Nakamura Y., Yamashita M., Suzuki T., Takamatsu K., Huang Y., Bae J., Yamashita S., Murata M., Yamada S. (2017). Green tea polyphenol epigallocatechin-3-gallate suppresses toll-like receptor 4 expression via upregulation of E3 ubiquitin-protein ligase RNF216. J. Biol. Chem..

[80] Matsumoto E., Kataoka S., Mukai Y., Sato M., Sato S. (2017). Green tea extract intake during lactation modified cardiac macrophage infiltration and AMP-activated protein kinase phosphorylation in weanling rats from undernourished mother during gestation and lactation. J. Dev. Orig. Health Dis..

[81] Clifford M.N., van der Hooft J.J., Crozier A. (2013). Human studies on the absorption, distribution, metabolism, and excretion of tea polyphenols. Am. J. Clin. Nutr..

[82] Zhang J., Nie S., Wang S. (2013). Nanoencapsulation enhances epigallocatechin-3-gallate stability and its antiatherogenic bioactivities in macrophages. J. Agric. Food Chem..

[83] Mena P., Bresciani L., Brindani N., Ludwig I.A., Pereira-Caro G., Angelino D., Llorach R., Calani L., Brighenti F., Clifford M.N. (2019). Phenyl-γ-valerolactones and phenylvaleric acids, the main colonic metabolites of flavan-3-ols: synthesis, analysis, bioavailability, and bioactivity. Nat. Prod. Rep..

[84] Pereira-Caro G., Moreno-Rojas J.M., Brindani N., Del Rio D., Lean M.E.J., Hara Y., Crozier A. (2017). Bioavailability of black tea theaflavins: Absorption, metabolism, and colonic catabolism. J. Agric. Food Chem..

[85] Imbe H., Sano H., Miyawaki M., Fujisawa R., Miyasato M., Nakatsuji F., Haseda F., Tanimoto K., Terasaki J., Maeda-Yamamoto M. (2016). “Benifuuki” green tea, containing O-methylated EGCG reduces serum low-density lipoprotein cholesterol and lectin-like oxidized low-density lipoprotein receptor-1 ligands containing apolipoprotein B: A double-blind, placebo-controlled randomized trial. J. Funct. Foods.

[86] Igarashi Y., Obara T., Ishikuro M., Matsubara H., Shigihara M., Metoki H., Kikuya M., Sameshima Y., Tachibana H., Maeda-Yamamotog M. (2017). Randomized controlled trial of the effects of consumption of ‘Yabukita’ or ‘Benifuuki’ encapsulated tea-powder on low-density lipoprotein cholesterol level and body weight. Food Nutr. Res..

[87] Venkatakrishnan K., Chiu H.F., Cheng J.C., Chang Y., Lu Y.Y., Han Y.C., Shen Y.C., Tsai K.S., Wang C.K. (2018). Comparative studies on the hypolipidemic, antioxidant and hepatoprotective activities of catechin-enriched green and oolong tea in a double-blind clinical trial. Food Funct..

[88] Orem A., Alasalvar C., Kural B.V., Yaman S., Orem C., Karadag A., Pelvan E., Zawistowski J. (2017). Cardio-protective effects of phytosterol-enriched functional black tea in mild hypercholesterolemia subjects. J. Funct. Foods.

[89] Wasilewski R., Ubara E.O., Klonizakis M. (2016). Assessing the effects of a short-term green tea intervention in skin microvascular function and oxygen tension in older and younger adults. Microvasc. Res..

[90] Nogueira L.D.P., Nogueira Neto J.F., Klein M.R.S.T., Sanjuliani A.F. (2017). Short-term effects of green tea on blood pressure, endothelial function, and metabolic profile in obese prehypertensive women: A crossover randomized clinical trial. J. Am. Coll. Nutr..

[91] De Jesús Romero-Prado M.M., Curiel-Beltrán J.A., Miramontes-Espino M.V., Cardona-Muñoz E.G., Rios-Arellano A., Balam-Salazar L.B. (2015). Dietary flavonoids added to pharmacological antihypertensive therapy are effective in improving blood pressure. Basic Clin. Pharmacol..

[92] Grassi D., Draijer R., Desideri G., Mulder T., Ferri C. (2015). Black tea lowers blood pressure and wave reflections in fasted and postprandial conditions in hypertensive patients: A randomised study. Nutrients.

[93] Grassi D., Draijer R., Schalkwijk C., Desideri G., D’Angeli A., Francavilla S., Mulder T., Ferri C. (2016). Black tea increases circulating endothelial progenitor cells and improves flow mediated dilatation counteracting deleterious effects from a fat load in hypertensive patients: A randomized controlled study. Nutrients.

[94] Lorenz M., Rauhut F., Hofer C., Gwosc S., Mueller E., Praeger D., Zimmermann B.F., Wernecke K., Baumann G., Stangl K. (2017). Tea-induced improvement of endothelial function in humans: No role for epigallocatechin gallate (EGCG). Sci. Rep..

[95] Sanguigni V., Manco M., Sorge R., Gnessi L., Francomano D. (2017). Natural antioxidant ice cream acutely reduces oxidative stress and improves vascular function and physical performance in healthy individuals. Nutrition.

[96] Dower J.I., Geleijnse J.M., Gijsbers L., Schalkwijk C., Kromhout D., Hollman P.C. (2015). Supplementation of the pure flavonoids epicatechin and quercetin affects some biomarkers of endothelial dysfunction and inflammation in (pre)hypertensive adults: A randomized double-blind, placebo-controlled, crossover trial. J. Nutr..

[97] Luximon-Ramma A., Bahorun T., Crozier A., Zbarsky V., Datla K.P., Dexter D.T., Aruoma O.I. (2005). Characterization of the antioxidant functions of flavonoids and proanthocyanidins in Mauritian black teas. Food Res. Int..

